# Anatomic and radiograph study of the persistence of Foramen of Huschke

**DOI:** 10.1016/S1808-8694(15)31273-8

**Published:** 2015-10-20

**Authors:** Rodrigo Costa Moreno, Israel Chilvarquer, Jorge E. Hayek, Paulo Isaias Seraidarian

**Affiliations:** 1 Dentist, major at Dental School, Universidade Ibirapuera. Intern, Discipline of Dental Radiology, Department of Stomathology, University of Sao Paulo.; 2 Master, Ph.D. and Full Professor, FOUSP; Post-graduation at University of Texas at San Antonio - USA, Faculty Professor, Discipline of Imaging, Universidade Ibirapuera.; 3 Master and Ph.D. in Mouth Diagnosis, FOUSP, Joint Professor of Imaging, Universidade Ibirapuera.; 4 Master and Ph.D. in Restorative Dental Practice, major in Buccomaxillofacial Prostheses, coordinator of the Master Program in Dental Prosthesis, PUC-MG, Professor responsible for the Discipline of Occlusion and TMJ, Universidade Ibirapuera-Unib, Assistant Professor, Department of Dental Prosthesis, Dental School, São José dos Campos/UNESP. Dental School, Universidade Ibirapuera.

**Keywords:** Huschke’s Foramen, panoramic, submental vertex, corrected sagittal linear temporal mandibular joint tomograph

## Abstract

**Aim:** the aim of this study is to assess and locate the Foramen of Huschke. **Study design:** anatomical. **Material and Method:** using contrast material like gutta-percha and barium sulfate, through extraoral radiographs, such as panoramic, submental vertex and corrected saggital linear Temporal Mandibular Joint tomograms in four skulls where we clinically checked the existence of foramen of Huschke. **Results:** The results proved that the foramen of Huschke can be observed in skulls submitted to contrast using radiographic techniques.

## INTRODUCTION

Known as foramen of Huschke, the opening of the developing tympanic ring (Gardner et al., 1967) was first described by Emil Huschke, in 1889, as being a structure present during embryological development in the tympanic portion of the temporal bone that is normally closed by the age of 5 years (Faig-Leite, 1998).

**Figure 1:**
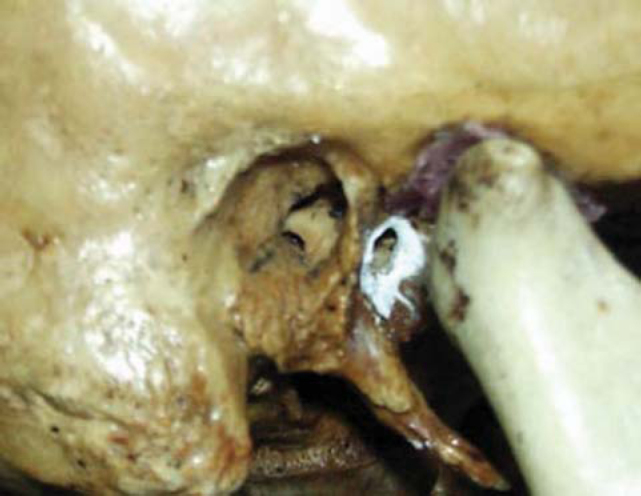
Foramen of Huschke evidenced with barium sulfate.

The persistence in adult subjects is known as an anatomical anomaly and it may be attributed to affections such as herniations of temporomandibular joint (TMJ), as well as otological problems in the external acoustic canal. Foramen of Huschke is located on the anterior wall of the external acoustic canal on the tympanic portion of the temporal bone, presenting a communication between the external acoustic canal and the mandibular fossa (Figure 1).

## OBJECTIVE

The purpose of the present study was to identify and locate the Foramen of Huschke through three different extraoral radiographic techniques in four dry skulls and to assess which radiological techniques may be used to identify the persistence of Foramen of Huschke.

## MATERIAL AND METHOD

To assess the presence of Foramen of Huschke in radiological exams, we used 4 dry human skulls, 3 of adult subjects who had persistence of Foramen of Huschke bilaterally and one subject aged about 4 years, in which the presence of the foramen was within the normal range. The skulls were submitted to extraoral techniques of panoramic x-ray (Figure 2A) with Orthopantomograph OP-100 (Instrumentarium Imaging - Tuusula, Finland), submental vertex technique (inverted Hirtz), and lateral linear tomography corrected for TMJ (Figure 2B) with dental tomographer Quint Sectograph (Denar Corporation, Anaheim, Calif. USA) and then new exams were performed using contrast material such as gutta-percha (sealing the foramen) and barium sulfate (on the foramen edges), to evidence the Foramen of Huschke on the x-rays.

**Figure 2A:**
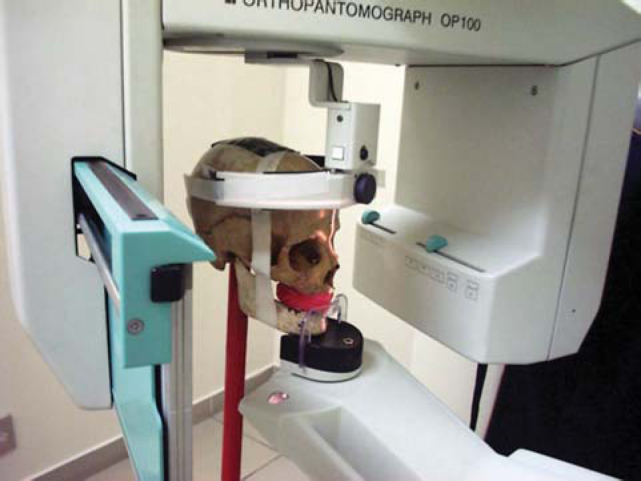
Panoramic technique.

**Figure 2B:**
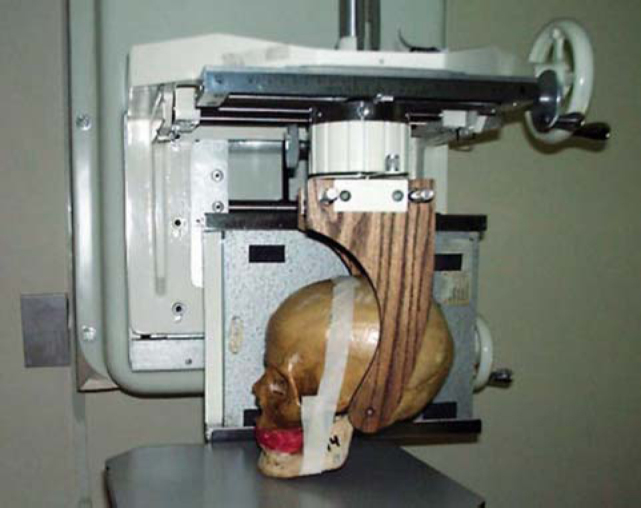
Corrected lateral CTs can.

After the execution of the exams, we compared the images with and without contrast material.

## RESULTS

The results found after the corrected lateral linear tomography without contrast material showed the presence of radiolucent area of elliptical format (Figure 3A).

**Figure 3A:**
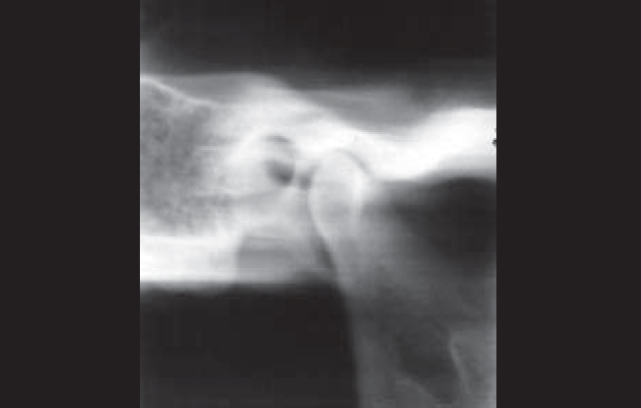
Corrected lateral CTs can - TMJ CT scan sections - section A without contrast material.

Even using barium sulfate, we observed radiolucent image of elliptical format with radiopaque contour (Figure 3B), and with gutta-percha, we detected the presence of radiopaque image of elliptical form (Figure 3C).

**Figure 3B:**
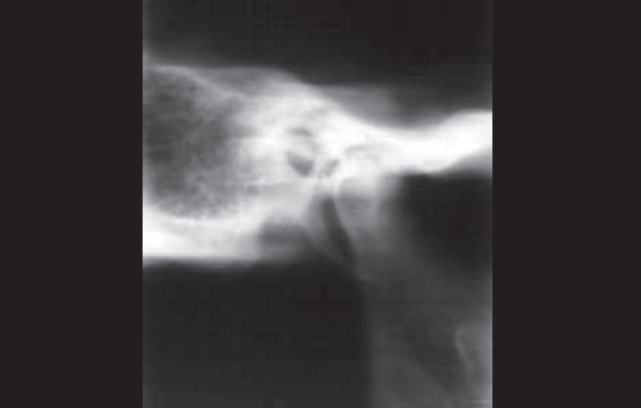
Corrected lateral CTs can - TMJ CT scan sections - section B with barium sulfate.

**Figure 3C:**
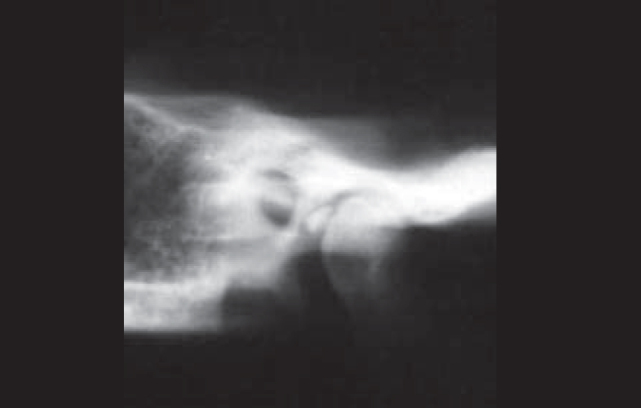
Corrected lateral CTs can - TMJ CT scan sections - section C with gutta-percha.

These images were located on the anterior wall of the external acoustic meatus on the tympanic portion of the temporal bone, presenting a communication between the external acoustic canal of the temporal bone tympanic portion, with the mandibular fossa of the temporal bone squamous portion.

**Figure 4:**
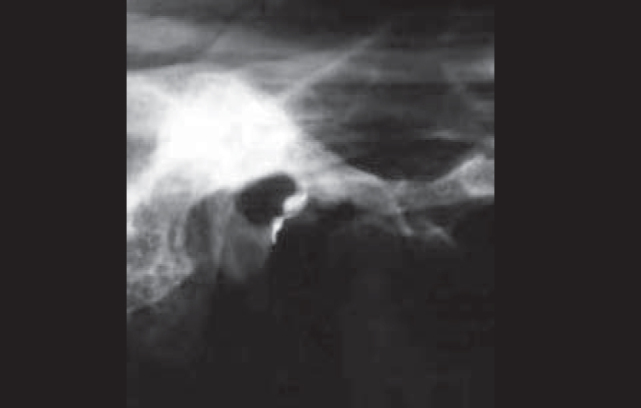
Panoramic technique, detail of the right TMJ region.

We observed that in the panoramic technique performed with barium sulfate, two radiopaque points presented communication between the external acoustic canal of the temporal bone tympanic portion, with the mandibular fossa of the temporal bone squamous portion (Figure 4).

**Figure 5:**
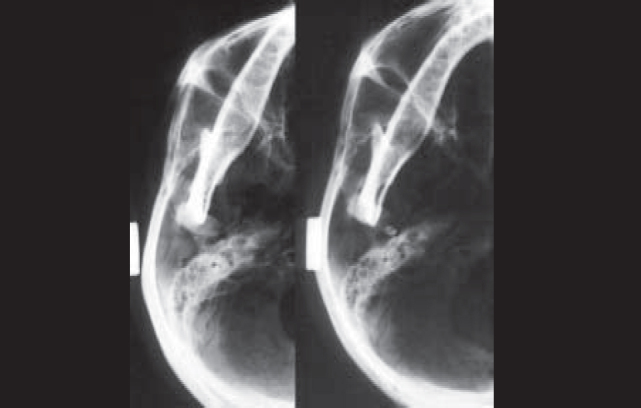
Submental vertex technique- detail of the right TMJ region; A. without contrast material, and B. with gutta-percha.

In the submental vertex technique, performed without contrast material (Figure 5A), we observed radiolucent image of elliptical form.

As to execution of images with gutta-percha, we observed elliptical radiopaque image (Figure 5B) and the images were located medially and posteriorly to mandible condyle, on the anterior wall of the external acoustic canal of the temporal bone tympanic portion.

The characteristics of the obtained radiographic images were the same for the four studied skulls.

## DISCUSSION

After the study of radiological images of four dry skulls we found the same results in the four skulls, observing the presence of Foramen of Huschke in the linear tomography technique. In submental vertex and panoramic techniques it was less evident as a result of the overlapping of bone structures.

Rosemberg and Graczik (1986) stated that the best radiological technique capable of identifying the structures that comprise the TMJ is corrected axial lateral tomogram, even though this radiological technique does not completely eliminate the overlapping of the TMJ region, because the structure that is located in the fulcrum of the rotation of the system will appear in more details and those that are below or above this point will seem to be blurred (Chilvarquer, 1995).

Owing to the overlapping of bone structures in the TMJ region, Holmlund et al. (1986) reported difficulty in examining TMJ through conventional radiograms. Therefore, they suggested that CT scan was the preferred method to avoid overlapping in this region.

Ali and Rubinstein (2000) detected in temporal bone CT scan a bone defect in the anterior aspect of the auditory canal, the defect known as Foramen of Huschke.

As to comparison of images initially obtained, with and without contrast (gutta-percha and barium sulfate), we observed that images described in the three radiological techniques are compatible with the anatomical location of Foramen of Huschke.

## CONCLUSION

Foramen of Huschke could be observed after its evidence with contrast material in radiological images of 4 dry skulls using extraoral, panoramic, submental vertex (inverted Hirtz) and TMJ corrected lateral linear tomogram techniques.

We concluded that among the used techniques, linear tomography proved to provide the best results and the literature recommends that CT scan is the best technique to visualize the Foramen of Huschke.
